# Reduction in hospitalisations for acute gastroenteritis-associated childhood seizures since introduction of rotavirus vaccination: a time-series and change-point analysis of hospital admissions in England

**DOI:** 10.1136/jech-2019-213055

**Published:** 2019-09-11

**Authors:** Daniel James Hungerford, Neil French, Miren Iturriza-Gómara, Jonathan M Read, Nigel A Cunliffe, Roberto Vivancos

**Affiliations:** 1 Centre for Global Vaccine Research, Institute of Infection and Global Health, University of Liverpool, Liverpool, UK; 2 NIHR Health Protection Research Unit in Gastrointestinal Infections, University of Liverpool, Liverpool, United Kingdom; 3 NIHR Health Protection Research Unit in Emerging and Zoonotic Infections, University of Liverpool, Liverpool, United Kingdom; 4 Field Service, National Infection Service, Public Health England, Liverpool, UK; 5 Tropical and Infectious Disease Unit, Royal Liverpool and Broadgreen University Hospitals NHS Trust, members of Liverpool Health Partners, Liverpool, United Kingdom; 6 Centre for Health Informatics, Computing and Statistics, Lancaster University, Faculty of Health and Medicine, Lancaster, UK; 7 Department of Microbiology, Alder Hey Children’s NHS Foundation Trust, members of Liverpool Health Partners, Liverpool, United Kingdom

**Keywords:** vaccination, time-series, diarrhoea, epidemiological methods, effectiveness

## Abstract

**Introduction:**

The incidence of severe childhood diarrhoea has fallen substantially following the introduction of rotavirus vaccine in the UK in July 2013. Since children with rotavirus infection may experience febrile and afebrile seizures, we evaluated the impact of rotavirus vaccination on seizure hospitalisations in children in England.

**Methods:**

Using data from Hospital Episode Statistics, we employed interrupted time-series analyses to assess changes in monthly hospital admissions for seizures among children aged <5 years from July 2000 to June 2017. Outcome measures comprised all seizures and febrile seizures, with and without a co-diagnosis of acute gastroenteritis (AGE). Models were adjusted for pneumococcal conjugate vaccine (PCV) introduction. Change-point analysis was used to independently identify step-changes in the time-series.

**Results:**

Among hospitalised children aged <5 years, the incidence of any seizures and febrile seizures with AGE decreased post-vaccine introduction by 23% (95% CI: 11% to 33%) and 31% (95% CI: 19% to 41%), respectively. For febrile seizures with AGE, a single change-point was identified in July 2013 (95% CI: June 2013 to December 2013). Reductions in seizure incidence were higher during the rotavirus season (49%, 95% CI: 37% to 58%) compared with out-of-season (13%, 95% CI: −4 to 28%) and showed no relation to PCV introduction. There were small reductions in any seizures with any co-diagnosis (4%, 95% CI: 0% to 8%) and in febrile seizures with any co-diagnosis (10%, 95% CI: 2% to 16%).

**Conclusion:**

Rotavirus vaccination has reduced hospitalisations for seizures associated with AGE in England, providing additional evidence of population-level impact of rotavirus vaccination on seizure incidence in high-income countries.

## Background

Prior to introduction of rotavirus vaccines into childhood immunisation programmes, rotavirus infection was the most common cause of severe gastroenteritis in children under 5 years, resulting in 40% of diarrhoea hospitalisations globally.^1^ In England, the human rotavirus vaccine Rotarix was introduced into its childhood immunisation schedule in July 2013, to be administered as two doses at 2 and 3 months of age.^2^ Vaccine uptake increased rapidly, reaching over 91% for one dose by February 2014 and 94.6% by July 2016.^3^ Since introduction, there have been large reductions in laboratory detections and hospitalisations for rotavirus gastroenteritis (RVGE),^4–6^ as well as significant reductions in less specific clinical outcomes such as hospitalisations for all-cause acute gastroenteritis (AGE).^4 5 7^

In addition to the commonly associated symptoms of diarrhoea and vomiting, rotavirus infection is also associated with childhood seizures.^8^ A large multicentre study in Canada showed that prior to rotavirus vaccine introduction, 7% of children hospitalised with laboratory-confirmed rotavirus infection suffered seizures.^9^ Several studies from different countries have assessed the impact and effectiveness of rotavirus vaccine introduction on seizures with conflicting findings. In two independent studies in the USA, risk of seizure emergency department (ED) attendance and admission was shown to be reduced by 21% and 24%, respectively, in those who were vaccinated.^10 11^ In Australia, rotavirus vaccination was 35%–38% effective at reducing febrile seizure ED attendances and admission in children under 2 years.^12^ However, population-level studies have found less convincing impact of rotavirus vaccination on seizure incidence. Analysis of a large US health database showed seizure hospital admission rates to be 1%–8% lower since rotavirus vaccine introduction, and a study from Spain showed a reduction of 16%–34%.^13 14^ However, recent studies from the UK, Portugal and Spain showed no measurable impact of rotavirus vaccination on seizure hospitalisations.^15–17^

Seizures associated with laboratory-confirmed rotavirus infection are likely to contribute a very small proportion of all seizures among hospitalised children. Therefore, detection of a population-level impact of rotavirus vaccination using all hospitalised seizures as an endpoint will be challenging. We therefore assessed population-level impact of rotavirus vaccination on hospitalised seizures in association with AGE in children in England, using interrupted time-series analysis, change-point analysis for multiple seizure related endpoints and further checks for plausible causation by analysing inrotavirus season and out-of-rotavirus season.

## Methods

### Data sources and case definitions

Aggregated hospital admission records were extracted from Hospital Episode Statistics (HES) accessed through Public Health England. HES is a record-based system maintained by National Health Service (NHS) digital, which contains information on all hospital admissions covering all NHS Trusts in England. Hospital episodes include codes for main diagnoses (primary diagnosis and 19 secondary diagnosis fields), which are assigned on discharge using the International Classification of Diseases, Tenth Revision (ICD-10). Seizures were defined using ICD-10 codes in primary or following diagnosis fields as ‘febrile convulsion’ (R56.0), ‘Other and unspecified convulsion’ (R56.8 and R56.9), ‘Convulsions of newborn’ (P90) and ‘Epilepsy’ (G40).

We could not include seizure hospitalisations with a co-diagnosis of rotavirus infection as an endpoint due to low numbers and low sensitivity of ICD-10-coded rotavirus in England (Heinsbroek *et al*, The impact of rotavirus vaccination on hospital pressures in a large paediatric hospital in the United Kingdom, NCT03271593).^18^ Because approximately 45% of AGE hospitalisations prior to rotavirus vaccine introduction were estimated to be associated with rotavirus infection, we used seizures with a co-diagnosis of AGE as our primary measure of vaccine impact on rotavirus-associated seizures.^1^

#### Study groups (seizure with any co-diagnosis or with age)

- Admission for febrile seizure (ICD-10 code: R56.0) with any co-diagnosis.
- Admission for seizure (ICD-10 code: R56.0, R56.8, R56.9, P90 or G40) with any co-diagnosis.
- Admissions for all-cause AGE (ICD-10 code: A00–A09, K52.9)^7 19^ and with a diagnosis of seizure (ICD-10 code: R56.0, R56.8, R56.9, P90, G40).
- Admissions for all-cause AGE (ICD-10 code: A00–A09, K52.9) and with a diagnosis of febrile seizure (ICD-10 code: R56.0)

We excluded any AGE admissions with a co-diagnosis of acute respiratory disease and pneumonia (ICD-10 codes: J0–J39, J860, J869, H65–H67, G001, A403, B953, M001).

In order to support attribution of a change in seizure incidence to rotavirus vaccine, we considered other widespread public health interventions that occurred during the study period. Since acute respiratory infections are associated with the majority of childhood seizures, and pneumococcal conjugate vaccines (PCVs) were introduced into the UK’s childhood immunisation schedule, we selected pneumonia-associated conditions as negative control endpoints. The seven-valent PCV (PCV7) was introduced in the UK in September 2006; PCV7 was replaced with the 13-valent PCV (PCV13) in April 2010, with doses at 8 and 16 weeks of age and a booster at 12–13 months of age.

#### Negative control groups (seizures with co-diagnosis of pneumonia)

ICD-10 codes for pneumonia were chosen based on PCV effectiveness and impact studies, local clinical audit and clinical assessment.^20^ For all negative control groups, admission episodes with a co-diagnosis of AGE were excluded.

- Admissions for seizure (ICD-10 code: R56.0, R56.8, R56.9, P90, G40) with a co-diagnosis of pneumonia (ICD-10 code: J12–J18, J860, J869, H65–H67, G001, A403, B953, M001).
- Admissions febrile seizure (ICD-10 code: R56.0) with a co-diagnosis of pneumonia (ICD-10 code: J12–J18, J860, J869, H65–H67, G001, A403, B953, M001).

### Statistical analyses

Statistical analyses were conducted in R V.3.5.1 (R Development Core Team, Vienna, Austria).

#### Rotavirus vaccine impact on seizure hospitalisations

We examined monthly hospital admissions for all study groups and negative control groups using an interrupted time-series design. First, to predict counterfactual numbers of admissions that would have been expected in the absence of vaccination for the vaccine period, we fitted generalised linear negative binomial models (to account for overdispersion in the data) to pre-vaccine introduction monthly counts, offset for age-specific population denominator and adjusting for a three-level categorical variable representing PCV7 and PCV13 introduction. We adjusted for seasonal trends and minor lag 1 autocorrelation by including seasonal harmonic terms and secular trends by including a linear term for surveillance year (July to June) as explanatory variables in the models. Second, to quantify the percentage change in monthly attendances/hospitalisations, we included all data pre-vaccine and post-vaccine introduction in a second model with a binary indicator variable denoting the post-vaccine period (1 July 2013 onwards). This second model also included the same terms to adjust for seasonal and secular trends, a three-level categorical variable representing PCV7 and PCV13 introduction and allowed the calculation of incidence rate ratios (IRRs). Percentage reduction was calculated as 100×(1-IRR).

The rotavirus season in the UK occurred consistently between the months of January and May in the pre-vaccine period, with a peak occurring in early to mid-March in most years.^21 22^ We therefore examined the specificity of ‘seizure/febrile seizure with a co-diagnosis of AGE’ in relation to rotavirus disease by stratifying by events that occurred inrotavirus season (January to May) and out-of-rotavirus season (June to December). For seasonal analysis, harmonic terms were replaced with a categorical term for month in the models. To examine the impact of rotavirus vaccination by age, we stratified our analyses by age group (<12 months, 12–23 months and 24–59 months).

#### Change-point

As an independent measure of potential vaccine impact, we used change-point analyses of a time-series.^23–26^ Unlike in interrupted time-series analysis, an intervention time point is not prespecified in change-point time-series analysis. Therefore, with fewer assumptions, change-point analysis allows the model to identify significant changes in the time-series and the timing of these changes. Using R package *strucchange*, we used linear regression adjusting for seasonal trends and secular trends to estimate change-points through minimising the residual sum of squares.^27^

## Results

### Hospitalised seizure incidence

**Figure 1:**
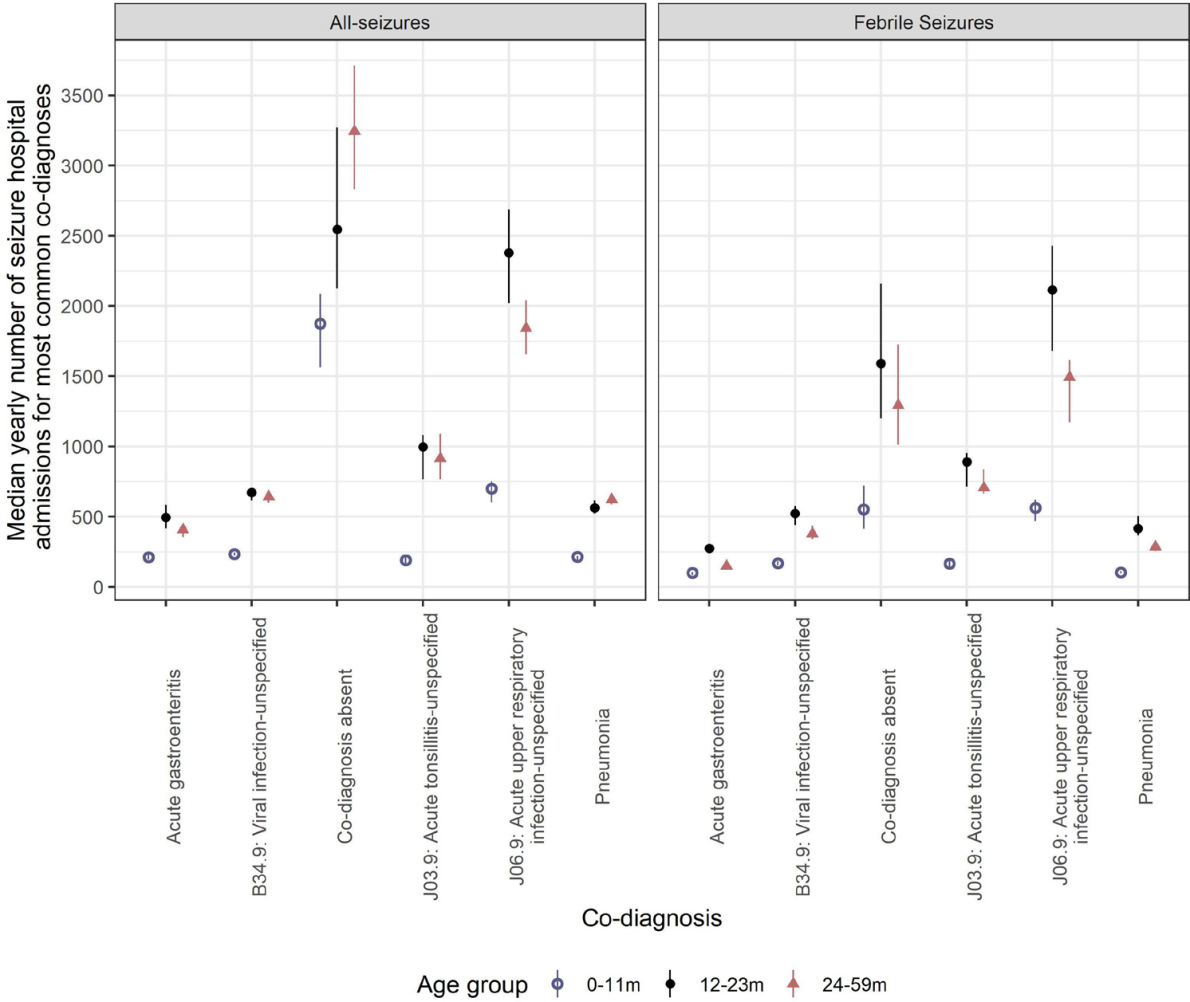
Median yearly number of seizure and febrile seizure hospital admissions and co-diagnoses, in children <5 years of age in England, by age group between July 2000 and June 2017. Error bars represent the IQR.

Prior to rotavirus vaccine introduction, there were 538 071 hospital admissions for all seizures and 246 699 admissions for febrile seizures in England between July 2000 and June 2017, at a median yearly rate of 84.7 and 38.8 per 10 000 population, respectively. The three most common co-diagnoses for febrile seizures were J06.9: acute upper respiratory infection-unspecified, J03.9: acute tonsillitis-unspecified and B34.9: viral infection-unspecified (online supplementary table 1; figure 1). Acute gastroenteritis was identified as a co-diagnosis in 3.5% (n=18 728) of all seizure hospitalisations and in 3.4% (n=8334) of febrile seizures. Children aged 24–59 months contributed 44% (n=237 512) of seizure hospitalisations. Children aged 12–23 months contributed 47.5% (n=117 222) of febrile seizure hospitalisations. Prior to rotavirus vaccine introduction, yearly rates of hospital admission for all seizures with any diagnosis were highest in 12–23 months olds (mean: 180.7 per 10 000 population), with a mean rate of 108.6 per 10 000 population in infants <12 months old (table 1). The rates of hospital admission were highest in 12–23 months olds across all study groups (table 1).

10.1136/jech-2019-213055.supp1 Supplementary data

**Table 1:** Changes in rates of hospital admissions for febrile and all seizures in England

Age group (months)	Mean yearly rate of hospital admission (per 10 000)			Percentage reduction in hospital admissions post-vaccine introduction (95% CI)†		
	Pre-rotavirus vaccination	Post-rotavirus vaccination (July 2013 to June 2017)				
	Observed	Observed	Expected*	PCV7	PCV 13	RV
All seizures						
Hospital admissions for all seizures with any co-diagnosis						
<12	108.6	107.8	104.9	−2 (−8 to 3)	1 (-7 to 9)	−4 (−8 to 0)
12–23	180.7	144.8	153.1	5 (−1 to 11)	13 (5 to 21)	7 (2 to 12)
24–59	75.2	71.8	75.1	1 (−7 to 8)	4 (−7 to 14)	4 (−2 to 9)
0–59	103.2	93.3	97.7	2 (−5 to 7)	7 (−1 to 15)	4 (0 to 8)
Hospital admissions for all seizures with AGE						
<12	3.7	2.6	3.9	−16 (−45 to 7)	−12 (−59 to 22)	24 (7 to 39)
12–23	8.5	5.6	9.8	−13 (−31 to 4)	−12 (−41 to 12)	33 (20 to 44)
24–59	2.1	2.1	2.6	−10 (−28 to 5)	−7 (−33 to 15)	11 (−4 to 23)
0–59	3.7	2.9	4.3	−13 (−30 to 2)	−9 (−34 to 12)	23 (11 to 33)
Hospital admissions for all seizures with pneumonia						
<12	3.4	2.8	2.3	−2 (−23 to 16)	−9 (−46 to 19)	−22 (−44 to −4)
12–23	9.8	7	7	8 (−5 to 19)	0 (−21 to 18)	3 (−8 to 14)
24–59	3.3	3.2	3.4	−3 (−17 to 10)	−9 (−34 to 12)	2 (−11 to 13)
0–59	4.6	3.9	3.9	1 (−9 to 11)	−5 (−23 to 10)	−1 (−10 to 8)
Febrile seizures						
Hospital admissions for febrile seizures with any co-diagnosis						
<12	36.4	25.8	27	13 (5 to 21)	20 (9 to 30)	10 (4 to 16)
12–23	119.3	80.3	86.2	5 (−3 to 13)	16 (5 to 26)	8 (2 to 14)
24–59	31.6	22.1	24	4 (−10 to 16)	12 (−7 to 27)	8 (−2 to 18)
0–59	50.2	34.4	37.6	5 (−5 to 14)	15 (2 to 26)	10 (2 to 16)
Hospital admissions for febrile seizures with AGE						
<12	1.7	0.9	1.5	−16 (−48 to 8)	−6 (−55 to 27)	34 (16 to 48)
12–23	4.7	2.2	4.1	−29 (−54 to −8)	−22 (−59 to 7)	33 (19 to 45)
24–59	0.8	0.5	0.7	−2 (−28 to 19)	15 (−20 to 39)	29 (12 to 43)
0–59	1.8	0.9	1.6	−21 (−42 to −2)	−8 (−38 to 16)	31 (19 to 41)
Hospital admissions for febrile seizures with pneumonia						
<12	1.8	1.3	1.1	−17 (−50 to 9)	−40 (−104 to 3)	−27 (−59 to −1)
12–23	7.5	4.7	4.6	7 (−8 to 19)	−1 (−25 to 18)	1 (−13 to 13)
24–59	1.7	1.2	1.2	9 (−10 to 25)	6 (−26 to 29)	8 (−8 to 22)
0–59	2.9	1.9	1.9	3 (−10 to 15)	−3 (−25 to 14)	1 (−10 to 11)

*Expected in the absence of rotavirus vaccination.

†Percentage change is calculated as 1−incidence rate ratio.

PCV7, seven-valent pneumococcal conjugate vaccine; PCV13, 13-valent pneumococcal conjugate vaccine; RV, rotavirus vaccine.

### Time-series of hospitalised seizures

#### With any co-diagnosis

**Figure 2:**
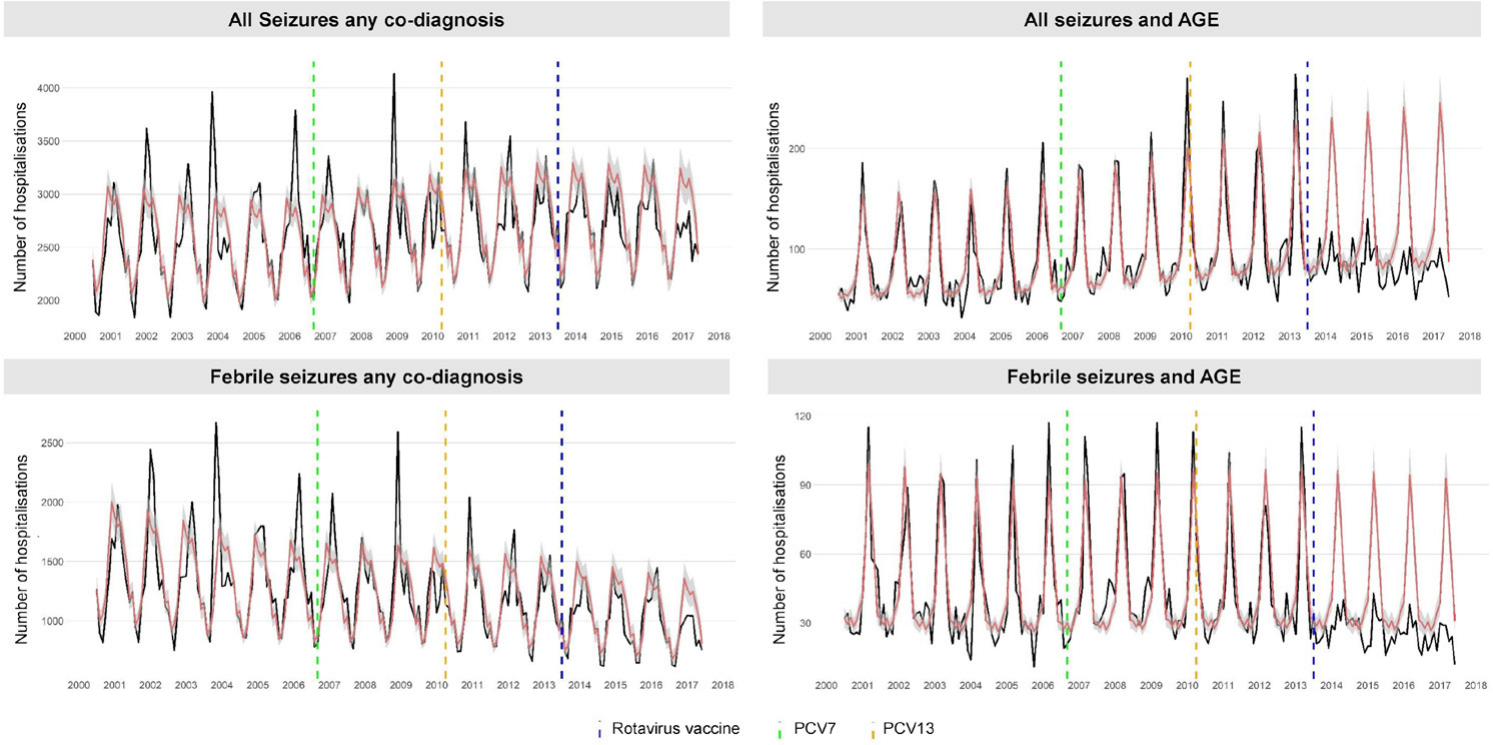
Monthly trends in hospitalisations for seizure in four study groups, for children <5 years of age in England, July 2000 to June 2017. Each analysis examines trends, including a comparison of observed incidence (black line) in England with expected incidence (red line) and associated 95% CIs (grey shaded area) in the absence of vaccination. Expected incidence and 95% CIs are based on predictions from regression models fitted to data for the period July 2000 to June 2013 for each outcome measure. The blue hashed line represents the introduction of rotavirus vaccine in the UK in July 2013, the green hashed line represents the introduction of the seven-valent pneumococcal conjugate vaccine (PCV7) in September 2006 and the yellow hashed line represents the replaced PCV7 with the 13-valent pneumococcal conjugate vaccine (PCV13) in the UK in April 2010. AGE, acute all-cause gastroenteritis.

Peak seasonality of all seizures with any co-diagnosis occurred between November and March each year. There was a year-on-year increasing trend for all seizures prior to rotavirus vaccine introduction (figure 2). In children 0–4 years of age, the incidence of seizure and febrile seizure hospitalisations reduced by 4% (95% CI: 0% to 8%; p=0.065) and 10% (95% CI: 2% to 16%; p=0.011), respectively, following rotavirus vaccination (table 1). For all seizures, this reduction was greatest in children aged 12–23 months (7%: 95% CI: 2% to 12%; p=0.005). There was also a reduction in febrile seizures in children aged <5 years following PCV13 introduction (16%: 95% CI: 5% to 26%; p=0.007). Change-points were not identified for these endpoints.

#### With AGE co-diagnosis

Peak seasonality of all seizures with a co-diagnosis of AGE occurred in March. Similar to all seizures with any co-diagnosis, hospitalisations for seizure associated with AGE increased year-on-year prior to rotavirus vaccination (figure 2). In children 0–4 years of age, the incidence of hospitalised seizures and febrile seizures with a co-diagnosis of AGE decreased post-vaccine introduction by 23% (95% CI: 11% to 33%; p<0.001) and 31% (95% CI: 19% to 41%), respectively. Reductions in seizure hospitalisation incidence were highest in children aged 12–23 months (33%: 95% CI: 20% to 44%; p<0.001) and lowest in children aged 24–59 months (11%: 95% CI: −4% to 23%; p=0.136). During the rotavirus season, seizure reductions (all seizures=43%: 95% CI: 32% to 52%; p<0.001 and febrile seizures=49%, 95% CI: 37% to 58%) were greater than out of season (all seizures=5%: 95% CI: −10% to 18%; p<0.001 and febrile seizures=13%, 95% CI: −4% to 28%). There were no observed changes in incidence of seizures with co-diagnosis of AGE following PCV7 and PCV13 introduction. Only a single change-point was identified, for all seizures with a co-diagnosis of AGE in April 2013 (95% CI: March 2013 to August 2013) and for febrile seizures with a co-diagnosis of AGE in July 2013 (95% CI: June 2013 to December 2013).

#### Negative control analysis

Hospitalised seizures with a co-diagnosis of pneumonia-associated conditions showed a seasonal peak between November and March (online supplementary figure 1). Prior to rotavirus vaccine introduction, pneumonia-associated seizure hospitalisations displayed a downward trend. There was no detectable impact of PCV7, PCV13 or rotavirus vaccine introduction on these endpoints and no change-point was identified (table 1).

## Discussion

We have demonstrated that in children under the age of 5 years, substantial decreases occurred in all-cause seizure and febrile seizure-coded hospitalisations with a co-diagnosis of AGE following rotavirus vaccine introduction. Our findings provide compelling evidence that rotavirus vaccination is responsible for these reductions. First, the magnitude of reduction for seizures with a co-diagnosis of AGE is consistent with previously reported reductions for all-cause hospitalised AGE.^4 7^ Second, the reduction in seizures with a co-diagnosis of AGE was substantially higher during the rotavirus season compared with out-of-season and was higher in younger, vaccine age-eligible children. Third, no impact of PCV7 and PCV13 was detected for seizures with a co-diagnosis of AGE. Fourth, change-point analysis identified a break-point at the time of the introduction of rotavirus vaccination in the UK immunisation schedule (July 2013). Lastly, the incidence of pneumonia-associated seizures did not fall following rotavirus vaccine introduction. Our findings are biologically plausible, since rotavirus infection is associated with fever leading to seizures, and rotavirus vaccination is highly (>80%) effective in reducing hospitalised RVGE in high-income settings.^28^ We also noted small reductions in all-cause seizures and febrile seizure-coded hospitalisations.

Our data support findings from the USA, Spain and Australia that reported reductions in all-cause hospital seizures following rotavirus vaccine introduction.^10 12–14^ However, subsequent studies conducted in Portugal and Spain failed to document a decline in population-level seizure incidence following vaccine introduction^16 17^; this may be explained by low (<50%) vaccine coverage and/or the small contribution of rotavirus to total seizure hospitalisations.^29 30^ Furthermore, a study from the UK that also failed to detect a reduction in total hospitalised seizure incidence following rotavirus vaccine introduction examined shorter pre-vaccination and post-vaccination time periods than our study, did not include a co-diagnosis of AGE or adjust for PCV introduction and employed an annual time-series model that does not account for in-year seasonal variations.^15^

We were unable to use rotavirus infection-related seizures as an endpoint because ICD-10 code A08.0 (rotaviral enteritis) is rarely used for laboratory-confirmed rotavirus, and therefore the number of A08.0 coded hospitalisations with a diagnosis of seizure was too few to analyse. As a proxy measure, we used seizures with a co-diagnosis of AGE and stratified our analysis by rotavirus season. Our analysis also provides clinicians with a quantified descriptive knowledge of the burden of seizures associated with AGE in the paediatric population in England. Our population-based study did not include individual vaccine status; however, the value of using these data in England is limited because of the high level of vaccine coverage (>94% for first dose and 89% for course completion).^3^ Also, because our study focused on hospitalisations, we were unable to investigate seizure incidence in the community. Finally, while the observed reduction in all-cause seizure incidence with any co-diagnosis following PCV13 introduction suggests some impact of PCV on seizure-associated lower respiratory tract infections, further subsequent reductions could be ascribed to rotavirus vaccination.

Our study confirms that rotavirus vaccination brings benefits beyond the cardinal symptoms of diarrhoea and vomiting that are associated with rotavirus disease. Quantifying the absolute population impact of rotavirus-associated seizures remains problematic because of non-specific outcome measures and the low relative burden of hospital inpatient rotavirus-associated seizures. Finally, our study highlights the importance of considering the base-line burden of disease when selecting outcome measures and the value of including multifaceted analytical approaches to provide confidence in findings from population-level impact evaluation.

**What is already known on this subject**

- In addition to symptoms of diarrhoea and vomiting, rotavirus infection may be accompanied by febrile and non-febrile seizures.
- Studies have reported a significant reduction in the individual risk of seizure hospitalisation following rotavirus vaccination in children <5 years of age.
- However, population-level impact of rotavirus vaccination on hospitalised seizure incidence has not been conclusively demonstrated.

**What this study adds**

- We demonstrate that the incidence of gastroenteritis-associated hospital seizures in England has fallen since rotavirus vaccine introduction.
- Two independent analytical approaches provide confidence that the observed reduction in seizure hospitalisations is attributable to rotavirus vaccination.
- Our study provides further, strong evidence that rotavirus vaccination results in health benefit beyond prevention of gastroenteritis.
