# Supplementary material for: Reduction in hospitalisations for acute gastroenteritis-associated childhood seizures since introduction of rotavirus vaccination: a time-series and change-point analysis of hospital admissions in England
Source: J Epidemiol Community Health. 2019 Sep 11;73(11):1020–5. doi: 10.1136/jech-2019-213055 (PMC6877709; doi:10.1136/jech-2019-213055)
Supplement: Supplementary data [file jech-2019-213055supp001.pdf]

Supplementary Materials

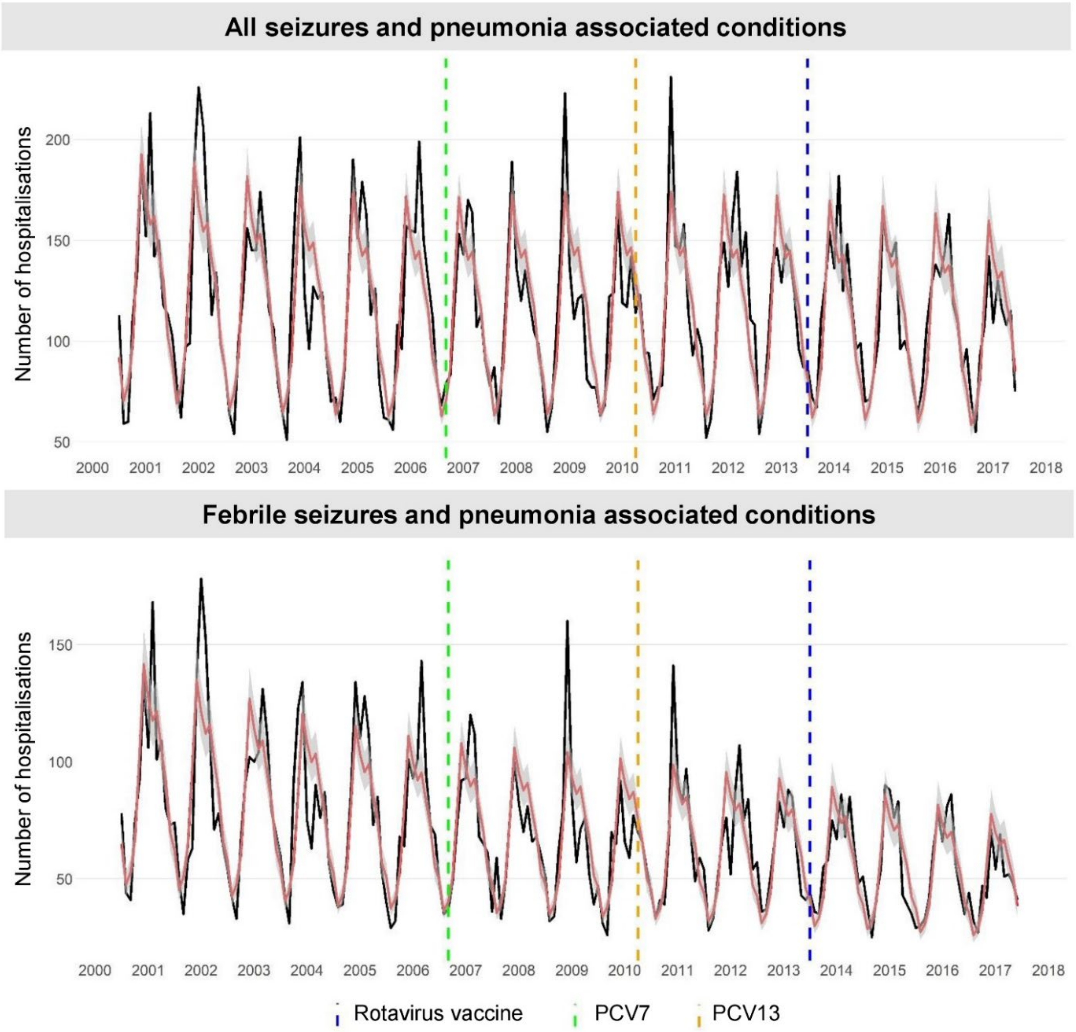

Supplementary Figure 1: Monthly trends in hospitalisations for validation groups for children <5 years of age in England, July 2000 to June 2017.

Each analysis examines trends, including a comparison of observed incidence (black line) in England with expected incidence (red line) and associated 95% confidence intervals (grey shaded area) in the absence of vaccination. Expected incidence and 95% confidence intervals are based on predictions from regression models fitted to data for the period July 2000 to June 2013 for each outcome measure. The blue hashed line represents the introduction of rotavirus vaccine in the UK in July 2013; the green hashed line represents the introduction of the 7 valent pneumococcal conjugate vaccine (PCV7) in September 2006; and, the yellow hashed line represents the replaced of PCV7 with the 13 valent pneumococcal conjugate vaccine (PCV13) in the UK in April 2010.

Supplementary Table 1: Number of seizure and febrile seizure hospital admissions and co-diagnoses, in children <5 years of age in England, between July 2000 and June 2017.

Co-diagnosis	Febrile Seizures	All-seizures
	**Yearly Median (IQR)**	**Yearly Median (IQR)**
**0-59 months of age**		
Acute gastroenteritis	534 (471 - 560)	1096 (963 - 1255)
Pneumonia	799 (722 - 928)	1370 (1339 - 1441)
J06.9: Acute upper respiratory infection-unspecified	4169 (3380 - 4620)	4925 (4303 - 5441)
J03.9: Acute tonsillitis-unspecified	1776 (1493 - 1971)	2070 (1673 - 2420)
B34.9: Viral infection-unspecified	1048 (981 - 1181)	1503 (1447 - 1587)
Co-diagnosis absent	3421 (2586 - 4537)	7606 (6465 - 9138)
Any co-diagnosis	15007 (13241 - 15977)	31909 (30878 - 32588)
**0-11 months of age**		
Acute gastroenteritis	99 (84 - 106)	211 (196 - 249)
Pneumonia	101 (97 - 112)	214 (189 - 220)
J06.9: Acute upper respiratory infection-unspecified	564 (468 - 621)	698 (600 - 750)
J03.9: Acute tonsillitis-unspecified	166 (144 - 192)	190 (154 - 223)
B34.9: Viral infection-unspecified	167 (152 - 184)	232 (222 - 244)
Co-diagnosis absent	552 (413 - 720.5)	1874 (1563.5 - 2085)
Any co-diagnosis	2242 (1993 - 2312)	7100 (6448 - 7208)
**12-23 months of age**		
Acute gastroenteritis	273 (251 - 294)	496 (415 - 584)
Pneumonia	416 (368 - 503)	563 (521 - 615)
J06.9: Acute upper respiratory infection-unspecified	2114 (1679 - 2427)	2379 (2018 - 2686)
J03.9: Acute tonsillitis-unspecified	891 (713 - 954)	996 (766 - 1080)
B34.9: Viral infection-unspecified	521 (439 - 577)	672 (614 - 678)
Co-diagnosis absent	1590 (1200 - 2159)	2545 (2124 - 3271)
Any co-diagnosis	7340 (6241 - 7772)	10827 (10641 - 11330)
**24-59 months of age**		
Acute gastroenteritis	149 (126 - 162)	407 (352 - 433)
Pneumonia	285 (256 - 323)	622 (587 - 630)
J06.9: Acute upper respiratory infection-unspecified	1491 (1170 - 1615)	1841 (1655 - 2040)
J03.9: Acute tonsillitis-unspecified	707 (663 - 836)	913 (764 - 1090)
B34.9: Viral infection-unspecified	378 (339 - 435)	642 (598 - 669)
Co-diagnosis absent	1292 (1013.5 - 1725.75)	3244.5 (2830 - 3712)
Any co-diagnosis	5389 (5007 - 5968)	13595 (13359 - 14753)

IQR: Interquartile range
